# Advances in biomarkers for optic neuritis and neuromyelitis optica spectrum disorders: a multi-omics perspective

**DOI:** 10.3389/fneur.2025.1559172

**Published:** 2025-05-06

**Authors:** Lidong Liu, Kai Guo, Dayong Yang

**Affiliations:** ^1^Department of Ophthalmology, The Affiliated Hospital of Inner Mongolia Medical University, Hohhot, China; ^2^Department of Neurology, Mayo Clinic, Rochester, MN, United States

**Keywords:** optic neuritis, neuromyelitis optica spectrum disorders, biomarker, multi-omics, AQP4, MOG

## Abstract

Optic neuritis (ON) and neuromyelitis optica spectrum disorders (NMOSD) are inflammatory neuro-ophthalmological conditions characterized by significant visual impairment and diverse clinical manifestations. Advances in diagnostic biomarkers have improved disease identification, including aquaporin-4 immunoglobulin G (AQP4-IgG) and myelin oligodendrocyte glycoprotein immunoglobulin G (MOG-IgG). However, some patients remain biomarker-negative, complicating differential diagnosis and personalized treatment. Multi-omics approaches have provided valuable insights into critical molecular pathways, novel biomarkers, and the shared and distinct features of ON and NMOSD. This review highlights recent advancements in biomarker research for ON and NMOSD, emphasizes the potential of multi-omics integration, identifies existing challenges, and proposes future research directions. These findings aim to enhance diagnostic accuracy, improve prognostic capabilities, and support the development of precision medicine strategies for ON and NMOSD.

## 1 Introduction

Optic neuritis (ON) and neuromyelitis optica spectrum disorders (NMOSD) are closely related neuro-ophthalmic diseases that present with acute vision loss and significantly affect patients' lives (1). ON is characterized by optic nerve inflammation and commonly manifests with symptoms such as vision loss, eye movement-associated pain, and color desaturation. While it is frequently associated with multiple sclerosis (MS), ON can also occur independently or as part of systemic autoimmune diseases (2). In contrast, NMOSD is a chronic inflammatory disorder primarily targeting the optic nerves and spinal cord. Its clinical presentation includes vision loss, confusion, weakness, and various central nervous system (CNS) symptoms (3). ON and NMOSD frequently involve optic nerve inflammation, making the differentiation between isolated ON, MS-related ON, and NMOSD-associated ON challenging based on clinical features alone (4). Furthermore, both ON and NMOSD share common immune-mediated inflammatory mechanisms and overlapping molecular and immunological pathways (5). Thus, a comprehensive analysis of the latest research progress in biomarkers for ON and NMOSD can systematically highlight the connections and differences between these two diseases, address existing research gaps, and propose key directions for future investigations, providing valuable support to researchers and clinicians.

Imaging techniques such as optical coherence tomography (OCT) and magnetic resonance imaging (MRI) are important in diagnosing ON and NMOSD. However, these tools lack the specificity to differentiate between these diseases (6). The identification of AQP4-IgG has revolutionized the diagnostic process for NMOSD, distinguishing it as a distinct entity separate from MS (7). Meanwhile, anti-AQP4 therapies have demonstrated significant clinical benefits in NMOSD, particularly in reducing relapse rates and enhancing patients' quality of life. Similarly, the MOG-IgG discovery has clarified the etiology of previously ambiguous ON and NMOSD cases (8). Despite these advancements, a substantial proportion of patients remain negative for AQP4-IgG and MOG-IgG. Therefore, there is a critical need to deepen the understanding of disease pathology and emphasize the development of additional biomarker-based diagnostic strategies to enhance differentiation and guide appropriate therapeutic interventions (9).

The advent of multi-omics—encompassing genomics, transcriptomics, proteomics, metabolomics, and epigenomics—has created unprecedented opportunities to unravel the complex biological networks underlying ON and NMOSD. Genomic studies have identified genetic susceptibilities associated with human leukocyte antigen (HLA) alleles and immune regulatory genes in NMOSD (10). Transcriptomic analyses have uncovered differentially expressed genes linked to disease activity in both diseases (11). Additionally, proteomic and metabolomic investigations have revealed protein and metabolic signatures indicative of inflammation and neuronal injury related to ON and NMOSD. Together, these multi-omics approaches facilitate the identification of potential biomarkers and enhance our understanding of disease mechanisms, paving the way for more precise and targeted therapeutic interventions.

In summary, this review consolidates current knowledge on ON and NMOSD and highlights recent advancements in biomarker research, focusing on multi-omics approaches. By integrating insights from various omics disciplines, the review seeks to (1) elucidate the shared and distinct pathophysiological features of ON and NMOSD; (2) evaluate established biomarkers for both ON and NMOSD; (3) explore recent developments in multi-omics-based biomarker discovery; and (4) identify existing gaps in current multi-omics research, while proposing future directions. This comprehensive approach aims to deepen our understanding of ON and NMOSD, ultimately advancing strategies for diagnosis, prognosis, and treatment of these complex disorders.

## 2 Pathophysiological features of ON and NMOSD

ON is primarily characterized by inflammation and demyelination of the optic nerve. Immune-mediated processes play a central role, with innate and adaptive immune responses contributing to tissue damage. Demyelination results from autoreactive T cells and macrophages targeting and decreasing myelin proteins, like myelin essential protein (MBP) and proteolipid protein (PLP) (12). This is accompanied by the activation of microglia, increased production of pro-inflammatory cytokines such as elevated interleukin-6 (IL-6), interleukin-17 (IL-17), and Interferon (IFN), and disruption of the blood-brain barrier (BBB) (13). Due to the loss of myelin, metabolic dysregulation, ischemic injury, and other processes, axonal damage and loss lead to interruption of visual signal transmission (14). At the same time, inflammation triggers the activation of astrocytes and microglia, forming glial scars to repair the damage. However, this process hinders nerve regeneration and further aggravates the pathological process. Isolated ON, independently of MS or NMOSD, may involve different immune mechanisms, such as viral infections or non-specific inflammation (15).

NMOSD is a distinct autoimmune condition primarily driven by AQP4-IgG, which targets the water channel protein aquaporin-4 expressed on astrocytes (16). The binding of AQP4-IgG leads to astrocytic injury through complement-dependent cytotoxicity (CDC) and antibody-dependent cellular cytotoxicity (ADCC), resulting in secondary demyelination and neuronal damage (17). Astrocytic damage is a defining characteristic that distinguishes NMOSD from MS (18). In addition to AQP4-IgG, some NMOSD patients test positive for MOG-IgG, which targets myelin-producing oligodendrocytes and contributes to inflammation and demyelination (19). MOG-IgG is associated with primary demyelination and preservation of astrocytes, differentiating this subgroup from AQP4-IgG-positive NMOSD. Key features of NMOSD pathophysiology also include disruption of the BBB, which facilitates immune cell and antibody entry into the CNS, and extensive complement-mediated cytotoxicity (20). Both ON and NMOSD involve immune-mediated inflammation leading to demyelination and axonal injury (21). Activation of autoreactive T cells and macrophages is central to both diseases. These immune cells infiltrate the optic nerve and surrounding CNS tissue. Disruption of the BBB is a common feature, facilitating immune cell and antibody entry into the CNS. The resultant inflammation triggers gliosis and long-term axonal degeneration, contributing to visual impairment and functional deficits. ON is frequently linked to MS in many cases, where autoreactive T cells predominantly target myelin proteins (22). NMOSD, on the other hand, is distinct due to its strong association with pathogenic autoantibodies that target astrocytes, initiating complement-dependent cytotoxicity and astrocyte loss.

## 3 Established biomarkers for ON and NMOSD

### 3.1 Established biomarkers for ON

#### 3.1.1 Structural imaging markers

Minakaran et al. demonstrated that OCT is a non-invasive imaging tool capable of quantifying retinal nerve fiber layer (RNFL) and ganglion cell-inner plexiform layer (GCIPL) thickness. Thinning of these layers reflects axonal and neuronal damage, making OCT a reliable marker for structural injury in ON (23). However, OCT lacks the specificity required to differentiate between various causes of ON, potentially delaying timely diagnosis and intervention (24). Magnetic resonance imaging (MRI) remains essential for detecting optic nerve inflammation and demyelination, with T2-weighted hyperintensities and gadolinium enhancement confirming active inflammation (25).

#### 3.1.2 Inflammatory markers

Elevated levels of IL-6, IL-17, and IFN-γ have been identified in patients with ON, indicating BBB disruption and heightened inflammatory activity (26, 27). Wullschleger et al. demonstrated that cerebrospinal fluid (CSF) IL-6 levels exceeding 10 pg/mL could exclude MS with 96% sensitivity, potentially aiding in distinguishing MS from other differential diagnoses (28). Additionally, tumor necrosis factor-alpha (TNF-α) is significantly elevated in ON, suggesting its role in demyelination and axonal damage by activating immune cells and inducing oligodendrocyte apoptosis (29). Increased levels of IFN-γ have also been reported, driving T-helper 1 (Th1) cell activation and amplifying inflammatory responses (30). Conversely, the anti-inflammatory cytokine interleukin-10 (IL-10) is reduced in ON patients, leading to inadequate immune regulation and exacerbated inflammation (31). Among specific biomarkers, AQP4-IgG is crucial in diagnosing ON, particularly in NMOSD-ON-associated cases. The study confirmed that high titers of AQP4-IgG correlate with bilateral, severe ON, relapse, and long-term visual impairment (32). In contrast, MOG-IgG is often linked to anterior optic nerve involvement, frequently presenting with optic disc swelling. Significantly, patients with MOG-IgG-positive ON generally experience better visual recovery than those with AQP4-IgG-positive ON (33). Despite their diagnostic value, AQP4-IgG and MOG-IgG lack sufficient specificity to differentiate among MS, NMOSD, and idiopathic ON. Furthermore, these biomarkers show limited sensitivity and are not reliable predictors of relapse risk, disease activity, or prognosis, thereby constraining their broader clinical utility (34).

### 3.2 Established biomarkers for NMOSD

#### 3.2.1 Aquaporin-4 immunoglobulin G

AQP4-IgG is the hallmark biomarker of NMOSD, detected in approximately 70%−80% of patients (35). This autoantibody targets aquaporin-4, leading to astrocytic injury, and CNS inflammation. AQP4-IgG testing has revolutionized NMOSD diagnostic criteria, enabling early diagnosis and treatment (16). Cell-based assays (CBAs) are the gold standard for AQP4-IgG testing due to their superior sensitivity and specificity compared to earlier methods such as enzyme-linked immunosorbent assays (ELISA) or immunofluorescence assays (IFA). Serum-based testing is particularly favored for its effectiveness and cost efficiency in NMOSD diagnosis (36). Flow cytometry (FCM), especially with automated platforms, has further improved testing accuracy, consistency, and minimized manual errors. Isobe et al. demonstrated that FCM achieves a higher positive detection rate (51.7%) for AQP4-IgG compared to IFA (41.4%) (37).

#### 3.2.2 Myelin oligodendrocyte glycoprotein immunoglobulin G

MOG-IgG is a critical biomarker for diagnosing NMOSD, especially in patients who test negative for AQP4-IgG. Shahd et al. demonstrated that 42% of AQP4-IgG-negative NMOSD patients were MOG-IgG positive, highlighting its diagnostic utility (38). The presence of MOG-IgG effectively differentiates myelin oligodendrocyte glycoprotein antibody disease (MOGAD) from AQP4-IgG-positive NMOSD and other CNS disorders such as MS. Moreover, MOG-IgG testing offers valuable prognostic insights, as persistent MOG-IgG positivity is linked to clinical relapses (39).

#### 3.2.3 Glial fibrillary acidic protein

Studies have shown that serum glial fibrillary acidic protein (GFAP) levels are significantly elevated in NMOSD patients during acute attacks and correlate with disability, consistent with antibody-mediated astrocyte pathophysiology (40). Furthermore, Schindler et al. confirmed that GFAP levels during remission in NMOSD may predict future disease activity (41).

#### 3.2.4 Complement activation markers

Lin et al. reported that complement C3 and C4 levels were significantly lower in NMOSD patients than those with MOGAD and healthy controls, reflecting the destruction of the BBB (42). Miyamoto et al. demonstrated elevated levels of complement biomarkers Ba and C5b-9 in NMOSD, indicating active complement system involvement (43). Asavapanumas et al. demonstrated that elevated serum and CSF levels of complement activation products correlate with disease severity and the risk of relapse, providing diagnostic insights and guiding therapeutic strategies (44).

#### 3.2.5 Cytokines and chemokines

Matsushita et al. confirmed that IL-17, IL-6, and C-C Motif Chemokine Ligand 19 (CCL19) levels in the CSF were significantly elevated during the NMOSD relapsing phase. Additionally, increased levels of CXCL8 and CXCL10 were observed in NMOSD patients (45). Elevated serum concentrations of IL-6 and endothelin-1 (ET-1) were positively correlated with disease severity (46). Higher levels of IL-4, IL-24, and CCL19 were associated with an increased risk of NMOSD in patient with positive AQP4 antibodies (47). Conversely, IL-10 demonstrated a protective role by mitigating inflammatory responses in NMOSD, while the low levels of IL-19 observed during relapses further suggested its potential protective effect in NMOSD patients (48). These suggest that specific cytokines and chemokines levels may serve as diagnostic markers for NMOSD, predicting disease severity and informing the development of personalized treatment strategies.

## 4 Advances in multi-omics biomarker discovery of ON and NMOSD

### 4.1 Multi-omics biomarkers in ON

#### 4.1.1 Genomics

Greta et al. demonstrated that STAT4 haplotypes G-G-A-C and C-T-A-T increase the risk of ON by up to 32.6-fold (49). Mostafa et al. revealed that the expression levels of interleukin-7 receptor (IL-7R) are significantly elevated in the peripheral blood and optic nerve tissue of ON patients (50). Additionally, Habek et al. confirmed that HLA-DRB1 variants may influence disease susceptibility, progression, and immune dysregulation in ON by modulating antigen presentation and T-cell responses. HLA-DRB1^*^15:01 has been strongly associated with ON cases (51).

#### 4.1.2 Transcriptomics

Transcriptomics offers a powerful tool for understanding the molecular basis of ON, with potential applications in diagnosis, prognosis, and treatment. Whole blood Transcriptomics analysis revealed that the gene expression profiles of patients with ON were significantly different compared with healthy controls. Seven hundred twenty-two differentially expressed genes were identified, with 377 exhibiting increased and 345 decreased expression. Quantitative PCR (qPCR) further validated the significant differential expression of SLPI, CR3, and ITGA4 among these genes, suggesting the potential role in early inflammatory responses (52). Additionally, Sørensen et al. reported that the CSF concentration of the CXCR3 ligand CXCL10 is selectively increased in ON patients, leading to the recruitment of CXCR3-positive cells to the subarachnoid space (53).

#### 4.1.3 Proteomics

AQP4-IgG and MOG-IgG are critical biomarkers for distinguishing subtypes of ON (54). Recent studies have explored their prognostic and therapeutic implications, revealing that AQP4-IgG positivity is associated with more severe clinical outcomes (55). Additionally, emerging research highlights the role of elevated neurofilament light chain (NfL) and heat shock proteins (HSPs) as indicators of axonal damage, stress response, and long-term prognosis in ON (56). Persistent NfL elevation has been shown to predict poor recovery of visual function and an increased likelihood of subsequent relapses (57). The application of proteomics in ON offers a novel perspective for understanding its pathological mechanisms, as well as for diagnosis and treatment.

#### 4.1.4 Metabolomics

Metabolomic studies have revealed significant alterations in energy metabolism in ON. Lactic and pyruvic acid have been confirmed as markers of optic nerve function (58). Another study reported elevated glutamate levels and reduced pyruvate concentrations in CSF, with glutamate excitotoxicity correlating with the severity of axonal injury and the potential for recovery (59). Lipid-related metabolism pathways, including sphingolipid metabolism and primary bile acid biosynthesis, have also been implicated in retinal neurodegeneration. Biomarkers such as taurine, taurochenodeoxycholic acid, taurocholic acid, sphingosine, and galactosylceramide have shown promise in optic nerve diseases (60). These findings provide valuable insights into the mechanisms of ON and may pave the way for future treatment strategies.

#### 4.1.5 Epigenomics

DNA methylation is crucial in normal neural functions, including synaptic plasticity, neuronal repair, learning, and memory. It has emerged as a potential clinical marker and therapeutic target. Abnormal DNA methylation is speculated to impact the development and function of the optic nerve, contributing to the onset and progression of ON (61). Cao et al. reported that HDAC4, HDAC7, KDM6A, and KDM5C exhibit potential as biomarkers for AQP4-associated ON, further underscoring the relevance of epigenetic regulation in the disease (62).

### 4.2 Multi-omics biomarkers in NMOSD

#### 4.2.1 Genomics

Shi et al. identified significant associations between the minor alleles of four STAT4 single nucleotide polymorphisms (SNPs) and an increased risk of NMOSD. These SNPs-rs7574865 T, rs10181656 G, rs10168266 T, and rs13426947 A-exhibited varying hazard ratios and confidence intervals, highlighting their potential role as genetic risk factors for NMOSD (63). Furthermore, the HLA-DQB1^*^0502 and other immune-related alleles have been shown to confer susceptibility to NMOSD, particularly in AQP4-IgG-positive cases (64). These findings underscore the importance of genetic factors in the pathogenesis of NMOSD and their potential use in risk stratification and personalized medicine.

#### 4.2.2 Transcriptomics

Researchers used next-generation sequencing (NGS) technology to observe distinct exosomal miRNA profiles in NMOSD patients compared to healthy controls (HCs). Chen et al. reported that hsa-miR-122-3p and hsa-miR-200a-5p were significantly upregulated in relapsing NMOSD patients and were explicitly expressed in NMOSD patients, with their expression levels positively correlating with disease severity (65). Noll et al. conducted a transcriptomic analysis and revealed the upregulation of IL-6 and astrocytic dysfunction-related genes as characteristic of NMOSD (66). In another study about AQP4-IgG-negative cases, RNA biomarkers associated with cytokine signaling, such as IL-10 and transforming growth factor beta-1 (TGF-β1), increased and correlated with immune cells' infiltration ratio (67). These biomarkers have shown promise in enhancing diagnostic accuracy, offering new avenues for personalized diagnostics and therapeutic intervention.

#### 4.2.3 Proteomics

Yandamuri et al. reported that CD16, CD56, and natural killer (NK) cells were significantly reduced in NMOSD samples, while levels of B cell activating factor (BAFF) were increased (68). Carta et al. found that Ago-Abs were detected in 6.7% of NMOSD patients and confirmed that these antibodies were associated with the myelitis phenotype and a more severe disease course (69). Shaygannejad et al. confirmed that increased levels of GFAP and complement activation products (C3a and C5b-9) in CSF and serum are specific biomarkers for NMOSD (70). Additionally, proteomic analysis of cerebrospinal fluid exosomes identified 442 proteins that can be used to distinguish NMO from MS (71). These Proteomic biomarkers are associated with severe disability and astrocyte damage, and can serve as predictors of disease progression and long-term disability.

#### 4.2.4 Metabolomics

Maxton et al. highlighted those levels of short-chain fatty acids (SCFAs), including acetate, propionate, and butyrate, were significantly reduced in NMOSD patients. Additionally, the study confirmed that elevated lactate and reduced pyruvate levels correlated with the disease's progression (72). These changes suggest a potential role for SCFAs in the pathogenesis of NMOSD. Similarly, Dias et al. reported that elevated LDL cholesterol levels are associated with inflammation and BBB damage, while reduced HDL cholesterol may contribute to CNS damage (73). These metabolic dysregulations are associated with astrocytic damage and may reflect ongoing neuroinflammatory processes, offering the potential for use in differential diagnosis and monitoring disease progression.

#### 4.2.5 Epigenomics

Epigenomic studies in NMOSD remain in the early stages. Epigenetic modifications, particularly DNA methylation, have emerged as potential contributors to neurodegenerative disorders and may hold promise for early disease detection and management (74). Additionally, noncoding RNAs—such as long non-coding RNAs, circular RNAs, and microRNAs—and histone modifications, including histone acetylation, have garnered significant attention for their potential roles in disease progression (75). Despite these encouraging findings, further in-depth studies are required to elucidate the underlying mechanisms and identify epigenetic markers that could facilitate accurate diagnosis and effective therapeutic interventions.

### 4.3 Integration of multi-omics data

The integration of multi-omics data has revolutionized the understanding of ON and NMOSD. Several distinct molecular pathways have been identified, which can enhance diagnostic accuracy and inform therapeutic strategies. Shaygannejad et al. demonstrated that combining genomic HLA associations with proteomic markers like GFAP significantly improves diagnostic specificity in NMOSD (70). Similarly, Moceri et al. reported that integrating metabolomic profiles, such as elevated lactate levels, with proteomic markers like NfL provides deeper insights into neuronal injury in ON (76). Advanced computational tools have further facilitated network analysis and pathway mapping, highlighting critical immune regulatory pathways such as IL-6 signaling across multiple omics layers (77). Bokhari et al. showed that combining AQP4-IgG serology with metabolomic and epigenomic data aids in diagnosing seronegative NMOSD cases, enhancing the sensitivity and specificity of biomarker-based diagnostics (78). Moreover, multi-omics approaches have demonstrated potential in predicting recovery trajectories and guiding treatment decisions based on individual molecular profiles, thereby improving ON's diagnostic and prognostic utility (79). In addition to refining diagnostic capabilities, multi-omics integration enables patient stratification based on molecular signatures, paving the way for personalized therapeutic approaches and bridging the gap between research and clinical application (80). Undoubtedly, the integration of multi-omics data has profoundly advanced the understanding and management of ON and NMOSD (81).

### 4.4 Multi-omics in seropositive and seronegative NMOSD

Elevated serum GFAP levels have been observed in AQP4-IgG-positive NMOSD patients compared to double-seronegative individuals (82). This suggests more pronounced astrocytic damage in seropositive cases. Higher concentrations of tau protein, indicative of neuronal injury, have been detected in seropositive NMOSD patients relative to seronegative counterparts (82). Increased levels of UCH-L1, a marker of neuronal damage, have been reported in seropositive NMOSD, differentiating them from seronegative patients (83). These findings underscore the potential of multi-omics approaches in elucidating the pathophysiological differences between seropositive and seronegative NMOSD, paving the way for more precise diagnostic and therapeutic strategies.

## 5 Gaps in current knowledge of multi-omics and future research directions

### 5.1 Current biomarker limitations and diagnostic gaps

Existing biomarkers, such as AQP4-IgG and MOG-IgG, fall short of capturing all cases of ON and NMOSD, particularly in negative patients, leading to a substantial diagnostic gap (84). Most studies have predominantly focused on well-established markers, often neglecting alternative pathways and novel candidates. This narrow scope limits the sensitivity and specificity of current diagnostic approaches and hinders the discovery of comprehensive biomarker panels. Additionally, the predominance of cross-sectional study designs restricts the ability to evaluate biomarker dynamics over time. This limitation reduces the predictive power of biomarkers in monitoring disease progression, relapse, remission, and therapeutic efficacy. Longitudinal studies are essential to address these gaps and enable the development of dynamic, time-sensitive diagnostic tools. Another critical issue is the underrepresentation of diverse ethnic groups in biomarker research. Most studies have been conducted in European and Asian populations, leaving significant gaps in understanding population-specific variations in biomarker expression. This lack of diversity diminishes the generalizability of findings and overlooks critical differences that could inform personalized diagnostic and therapeutic strategies (85).

### 5.2 Challenges in multi-omics integration and clinical translation

A significant challenge in multi-omics research lies in effectively integrating data across various omics levels. The high dimensionality and heterogeneity of multi-omics datasets complicate integrated analyses, further hampered by the absence of standardized protocols, making it challenging to select the most appropriate analytical methods (86). Although network inference and feature selection techniques have been proposed, these methods predominantly focus on integrating two omics levels rather than achieving comprehensive multi-level integration. Moreover, the extensive diversity of tools and methods available in the field has contributed to the lack of standardized guidelines, particularly in defining and classifying data integration strategies into early, intermediate, and late-stage approaches (87). The rapid advancement of single-cell multi-omics technologies has introduced additional complexities, particularly in integrating multi-omics data at the single-cell level (3). These challenges underscore the urgent need for developing robust, standardized frameworks to facilitate effective multi-omics data integration, enabling more comprehensive insights into complex biological systems. Although no standardized algorithm currently exists, emerging computational tools and predictive models hold promise for guiding the development of future diagnostic algorithms.

### 5.3 Future research directions

It is important to note that many of these biomarkers are in the discovery or early validation stages, and current evidence remains insufficient to establish them as definitive diagnostic tools. Future research should prioritize identifying novel biomarkers, including autoantibodies, non-coding RNAs, and cell-based assays, to broaden the range of diagnostic and prognostic tools. Large-scale, multi-center longitudinal studies are crucial for capturing the temporal dynamics of biomarker expression and their associations with disease progression and treatment efficacy (88). At the same time, efforts should focus on developing a unified framework for integrating multi-omics data to address challenges related to high dimensionality and data heterogeneity. Establishing standardized tools, benchmarks, and best practice guidelines will improve multi-omics research's reproducibility, scalability, and reliability. Additionally, developing more user-friendly techniques applicable in clinical settings is essential. These advancements can significantly enhance personalized medicine approaches and improve outcomes for complex diseases.

## 6 Conclusion

Biomarkers such as AQP4-IgG and MOG-IgG have been instrumental in diagnosing and treating ON and NMOSD, yet they still face issues with specificity and sensitivity. Integrating multi-omics approaches has significantly advanced our understanding of these diseases by identifying critical pathways, improving diagnostics, and uncovering potential therapeutic strategies. Multi-omics holds excellent promise and warrants further in-depth investigation. However, challenges remain, including small sample sizes and the lack of standardized methodologies. Future efforts should focus on identifying new biomarkers, conducting large-scale and multicenter studies, and ensuring the rational application of multi-omics. It will significantly improve the diagnosis, treatment, and prognosis of patients with ON and NMOSD.
